# Transcriptomics-based investigation of molecular mechanisms underlying synergistic antimicrobial effects of AgNPs and Domiphen on the human fungal pathogen *Aspergillus fumigatus*

**DOI:** 10.3389/fmicb.2023.1089267

**Published:** 2023-02-01

**Authors:** Wenlong Du, Ruolin Xu, Zhiqiang He, Huan Yang, Yufan Gu, Yi Liu

**Affiliations:** ^1^Department of Bioinformatics, School of Life Sciences, Xuzhou Medical University, Xuzhou, Jiangsu, China; ^2^Department of Biophysics, School of Life Sciences, Xuzhou Medical University, Xuzhou, Jiangsu, China

**Keywords:** *Aspergillus fumigatus*, RNA-Seq, AgNPs, domiphen, synergistic antimicrobial effects, fungi, antimicrobials

## Abstract

Critically ill patients have higher risk of serious fungal infections, such as invasive aspergillosis (IA) which is mainly caused by the human fungal pathogen *Aspergillus fumigatus*. Triazole drugs are the primary therapeutic agents for the first-line treatment of IA, which could easily cause drug resistance problems. Here, we assess the potential of AgNPs synthesized with *Artemisia argyi* leaf extract and domiphen as new antifungal agents to produce synergistic antimicrobial effects on *Aspergillus fumigatus*, and dissect possible molecular mechanisms of action. Plate inoculation assays combined with drug susceptibility test and cytotoxicity test showed that the combination of AgNPs and domiphen has synergistic antimicrobial effects on *A. fumigatus* with low cytotoxicity. Gene Ontology (GO) enrichment analysis showed that AgNPs and domiphen inhibit the growth of *A. fumigatus* by suppressing nitrate assimilation, and purine nucleobase metabolic process and amino acid transmembrane transport, respectively. When the two drugs are combined, AgNPs has epistatic effects on domiphen. Moreover, the combination of AgNPs and domiphen primarily influence secondary metabolites biosynthesis, steroid biosynthesis and nucleotide sugar metabolism of *A. fumigatus* via Kyoto Encyclopedia of Genes and Genomes (KEGG) enrichment analysis. Furthermore, protein–protein interactions (PPI) analysis combined with validation experiments showed that the combination of AgNPs and domiphen could enhance the expression of copper transporter and inhibit nitrogen source metabolism. In addition, the synergistic antimicrobial effects could be enhanced or eliminated depending on exogenous addition of copper and nitrogen source, respectively. Taken together, the results of this study provide a theoretical basis and a new strategy for the treatment of IA.

## 1. Introduction

In today’s medical environment, prevention and disinfection are increasingly important. Pathogenic microorganisms have caused serious damage to human life, health and property safety (Siscar-Lewin et al., 2019; Leitao, 2020; Simoes and Antunes, 2021). *Aspergillus fumigatus* is a widely existing pathogenic fungus in the natural environment (Du et al., 2021b). The airborne conidia produced by *Aspergillus fumigatus* could be inhaled into the terminal respiratory tract and cause respiratory diseases (Abad et al., 2010; van de Veerdonk et al., 2017; Du et al., 2021a). IA is the most common invasive fungal infection worldwide, and it has become the main cause of death for individuals with low immunity (such as patients after transplantation, major surgery, pulmonary fibrosis and chronic infection, etc.) (Cadena et al., 2016, 2021; Hsu et al., 2022). *A. fumigatus* is the main pathogen causing IA (Karthaus and Buchheidt, 2013). Triazole drugs are the primary therapeutic agents for the first-line treatment of IA (Roemer and Krysan, 2014; Esquivel et al., 2015), which could easily cause drug resistance problems. Thus, it is indispensable to develop new drugs to combat *A. fumigatus*. We previously reported that combination of AgNPs and domiphen is antimicrobial against biofilms of common pathogens (Hu et al., 2021). Therefore, this study aimed to discuss the potential of combining AgNPs and domiphen as new antifungal agents against the human fungal pathogen *A. fumigatus* and possible molecular mechanisms of action.

Currently, new antimicrobial drugs and drug delivery systems are developed based on nanotechnology with a strong focus on metal-based systems (Cu, Ag, Zn) (Minakshi et al., 2019). Nanotechnology is the most promising emerging technology in the 21st century, which is widely applied in information, biology, medicine, chemical industry and other fields, with huge market potential (Ehdaie, 2007; Barreto et al., 2011). The particle size of silver nanoparticles is mostly between 25–100 nm, which has strong inhibitory and killing effects on dozens of pathogenic microorganisms such as *Escherichia coli*, *Neisseria gonorrhoeae* and *Chlamydia trachomatis*, and is not easy to develop drug resistance (Chen and Schluesener, 2008). The emergence of nanotechnology has made a qualitative leap in the antimicrobial ability of silver at the nanoscale. Very little nanosilver could produce strong antimicrobial effects (Abram and Fromm, 2020). It could kill many kinds of microorganisms in minutes with broad spectrum at relatively low doses that are well tolerated in humans, which opens up a broad prospect for the wide application of nanosilver to antimicrobials, and it is a new generation of natural antimicrobial agent (Abram and Fromm, 2020). Here, we used *A. argyi* leaf as reducing and stabilizing agents for reducing AgNO_3_ to AgNPs, which could be comparatively safe and eco-friendly (Hu et al., 2021). However, a heavy use of AgNPs could cause its antimicrobial effects to be reduced by precipitation and aggregation (Hu et al., 2021). On the contrary, domiphen, a cationic surface active fungicide with broad spectrum, could be used for the adjuvant treatment of oral, throat, skin and wound infections and disinfection of surgical instruments (Fumagalli et al., 2018; Long et al., 2021). Domiphen is easy to be adsorbed by microbes and produce strong antimicrobial effects with low toxicity and doses, which could fully disperse AgNPs when the two agents are combined (Hu et al., 2021). Thus, we supposed that AgNPs and domiphen may have synergistic antimicrobial effects.

In this study, we aimed to explore the synergistic antimicrobial mechanism of AgNPs combined with domiphen against the pathogenic fungus *A. fumigatus* and to find the functional genes and potential drug targets affected by AgNPs and domiphen on the basis of RNA-sequencing (RNA-seq). We analyzed specific reasons especially copper and nitrogen source for synergistic antimicrobial effects of AgNPs and domiphen on *A. fumigatus* through exploring the influence of AgNPs, domiphen and their combination on gene expression of pathogenic fungus *A. fumigatus* combined with validation experiments. Until now, there have been no reports that combination of AgNPs and domiphen as new antifungal agents could combat *A. fumigatus*. The results of this study provide a theoretical basis and a new strategy for the treatment of IA.

## 2. Materials and methods

### 2.1. Reagents, strains, and media

*A. fumigatus* (A1161) was obtained from Professor Ling Lu’s lab (Jiang et al., 2014). AgNO_3_ (99.8%) and domiphen (97%) were purchased from Sinopharm Chemical Reagent Co., Ltd. (China) and Shanghai Yien Chemical Technology Co., Ltd. (China), respectively. *Artemisia argyi* leaf extract and glucose were purchased from Xi’an Runxue Bio-technology Co., Ltd. (China) and Aladdin Reagent Co., Ltd. (China), respectively. Agar and RPMI-1640 were purchased from Xuzhou Microcomande Biological Engineering Co., Ltd. (China) and Shanghai Sigma-Aldrich Trading Co., Ltd. (China), respectively. Yeast extract, ZnSO_4_·7H_2_O, H_3_BO_3_, MnCl_2_·4H_2_O, FeSO_4_·7H_2_O, CoCl_2_·5H_2_O, CuSO_4_·5H_2_O, (NH_4_)6Mo_7_O_24_·4H_2_O, Na_4_EDTA, NaNO_3_, KCl, CuCl_2_ ammonium tartrate (C_4_H_12_N_2_O_6_), MgSO_4_·7H_2_O and KH_2_PO_4_ were purchased from Shanghai Maclin Biochemical Technology Co., Ltd. (China). For RNA-seq, *A. fumigatus* strains were grown on liquid MM (minimal medium), containing 1% glucose, trace elements and 50 ml L^−1^ 20 × salt, pH 6.5. For the plate inoculation tests, *A. fumigatus* strains were grown on rich medium-YAG (yeast extract, agar and glucose), containing 2% glucose, trace elements, 0.5% yeast extract and 2% agar (Du et al., 2021b).

### 2.2. Preparation of AgNPs

The extract of *A. argyi* leaf was used as reducing agents. The obtained 1,000 μg ml^−1^ silver solution was filtered by 0.22 μm microporous membrane, and the particle size was about 77.6 nm measured by particle size analyzer (Hu et al., 2021). Briefly, 8.5 mg of AgNO_3_ and 10 mg of *A. argyi* leaf extract were dissolved in 5 ml distilled water and bathed in 100°C boiling water for 40 min to prepare 1,000 μg ml^−1^ AgNPs solution.

### 2.3. Drug dilution

Appropriate sterile distilled water was used for drug concentration dilution of AgNPs and domiphen solution. According to CLSI M-38A2 (Clinical and Laboratory Standards Institute) (Espinel-Ingroff et al., 2011), two-fold dilution method was used for drug concentration dilution.

### 2.4. Plate inoculation test

1 × 10^5^ CFU/ml, 1 × 10^6^ CFU/ml and 1 × 10^7^ CFU/ml of *A. fumigatus* conidia suspension were mixed with a vortex mixer for 20 s. Then, 2 μl of *A. fumigatus* conidia suspension was absorbed with a pipette gun and inoculated stably on the plate to form suspension droplets with a diameter of about 2 mm, which were placed in an incubator at 37°C, incubated for 36–48 h and to be photographed.

### 2.5. Single drug susceptibility test

Single drug susceptibility test was performed according to broth microdilution method and CLSI document M38-A2 (Espinel-Ingroff et al., 2011). Briefly, the same numbers of conidia (1 × 10^5^ conidia) were diluted into the RPMI-1640 medium containing gradient diluent antifungal drugs and transferred into 96-well plates and cultured at 35°C for 48 h (Li et al., 2020).

### 2.6. Combined drug susceptibility test

The microcheckerboard dilution method was used to determine the synergistic MICs of AgNPs and domiphen. *A. fumigatus* solution was prepared with 0.5 McTurbidite ratio (1 × 10^8^ CFU/ml) and diluted 100 times with sterile distilled water (1 × 10^6^ CFU/ml). AgNPs and domiphen were diluted with RPMI-1640. We used 1/8 MIC as a minimum unit to generate a series of concentration gradients from 1/8 MIC to 2 MIC. For the combined drug susceptibility test with copper and nitrogen source, 1 mM CuCl_2_ and 1 mM ammonium tartrate were added to RPMI-1640 media, respectively. Then 100 μl of fungal solution was added to the sterile 96-well plates, so that the final fungal inoculation amount was 5 × 10^5^ CFU/ml, and transferred into 96-well plates and cultured at 35°C for 48 h. The selected concentrations of agents for MIC test in 5 ml tubes were performed according to the MIC results in 96-well plates. The minimum drug concentration without fungal growth (obvious clarity) was denoted as MIC’. FIC calculation uses the following equation:


$$\begin{array}{c} \mathrm{FIC}={\mathrm{MIC}}^{'}\;\left(\mathrm{AgNPs}\right)/\mathrm{MIC}\;\left(\mathrm{AgNPs}\right)\\ +{\mathrm{MIC}}^{'}\;\left(\mathrm{Domiphen}\right)/\mathrm{MIC}\;\left(\mathrm{Domiphen}\right) \end{array}$$

FIC ≤ 0.5, 0.5 < FIC ≤ 1, 1 < FIC < 2 and FIC ≥ 2 represent synergistic effects, additive effects, independent effects and antagonistic effects, respectively.

### 2.7. Preparation of RNA-seq samples

2 × 10^7^ *A. fumigatus* wild-type conidia were inoculated into 100 ml MM liquid medium at 37°C and 220 rpm for 18 h, and then 1 μg ml^−1^ AgNPs, 2 μg ml^−1^ domiphen or both drugs were added for shaking of 1 h, respectively. The control group was not treated with drugs. Then the mycelia were filtered and collected with gauze, quick-frozen with liquid nitrogen, and stored at-80°C for three parallel repeats.

### 2.8. RNA extraction and quality control

Total RNA was extracted from the samples using TRIzol® Reagent according the manufacturer’s instructions (Invitrogen, CA, United States) and genomic DNA was removed using DNase I (TaKara, Japan). RNA degradation and contamination were monitored on 1% agarose gels. Then RNA quality was determined by 2,100 Bioanalyser (Agilent Technologies, Germany) and quantified using the ND-2000 (NanoDrop Technologies, Wilmington, DE, USA). Only high-quality RNA sample (OD260/280 = 1.8 ~ 2.2, OD260/230 ≥ 2.0, RIN ≥ 8.0, 28S:18S ≥ 1.0, > 1 μg) was used to construct sequencing library.

### 2.9. Transcriptomics profiling

RNA purification, reverse transcription, library construction and sequencing were performed at Shanghai Majorbio Bio-pharm Biotechnology Co., Ltd. (Shanghai, China) according to the manufacturer’s instructions (Illumina, San Diego, CA). The transcriptome library was prepared following TruSeq™ RNA sample preparation Kit from Illumina (San Diego, CA) using 1 μg of total RNA. Shortly, messenger RNA was isolated according to polyA selection method by oligo (dT) beads and then fragmented by the sharding buffer firstly. Secondly double-stranded cDNA was synthesized using a SuperScript double-stranded cDNA synthesis kit (Invitrogen, CA) with random hexamer primers (Illumina). Then the synthesized cDNA was end-repaired, phosphorylated and ‘A’ base addition according to Illumina’s library construction protocol. Libraries were size selected for cDNA target fragments of 300 bp on 2% Low Range Ultra Agarose followed by PCR amplified using Phusion DNA polymerase (NEB) for 15 PCR cycles. After quantified by TBS380, paired-end RNA-seq sequencing library was sequenced with the Illumina NovaSeq 6,000 sequencer (2 × 150 bp read length). The raw paired end reads were trimmed and quality controlled by fastp-v0.19.4^1^ with default parameters (Chen et al., 2018). Then clean reads were separately aligned to reference genome with orientation mode using HISAT-v2.2.1^2^ software (Kim et al., 2015). The mapped reads of each sample were assembled by StringTie-v2.2.0^3^ in a reference-based approach (Pertea et al., 2015).

### 2.10. Differential expression analysis and functional enrichment

**Figure 1:**
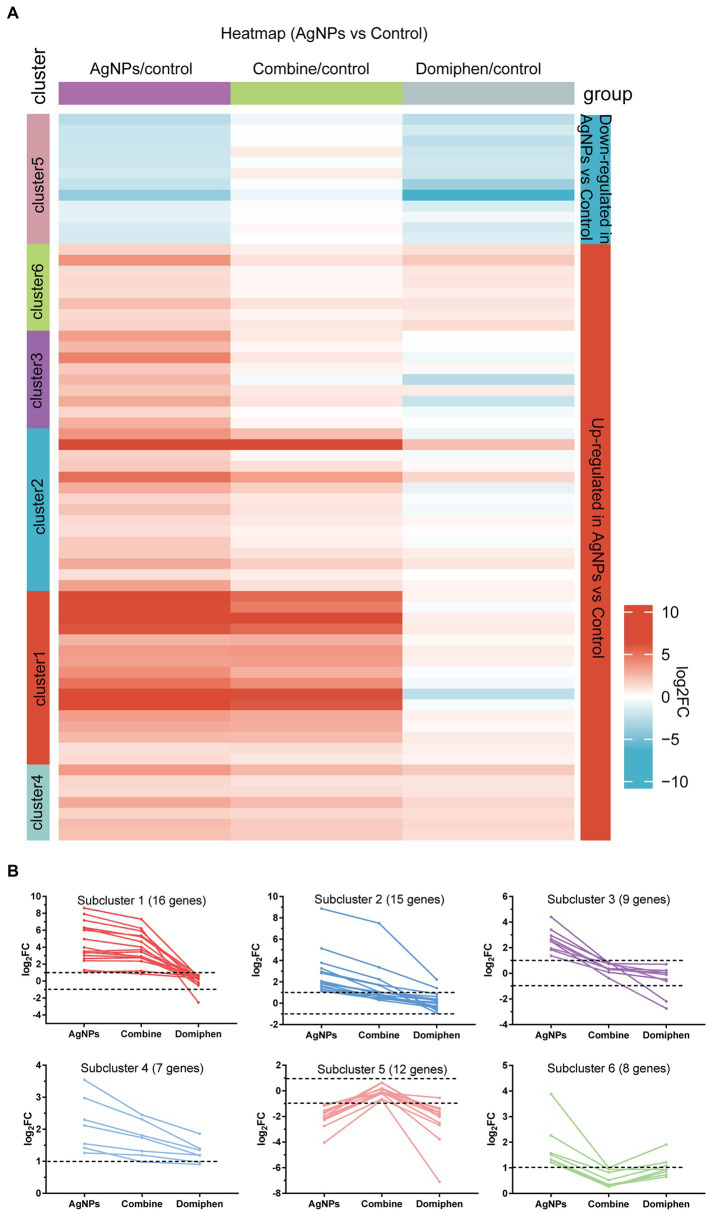
Transcriptional response of *A. fumigatus* to AgNPs. **(A)** Heatmap showing the changes in expression of the genes significantly downregulated (blue; the upper) and upregulated (red; the bottom) after 1 h treatment of AgNPs. Each column represents a group, and each row represents a DEG. The color represents the expression value (log_2_FC, the average from three parallel experiments) of the gene in each group. Red and blue represent log_2_FC > 0 and log_2_FC < 0, respectively. “1–6” indicate that multiple expression clusters could be differentiated. **(B)** The average expression (log_2_FC) of multiple genes.

**Figure 2:**
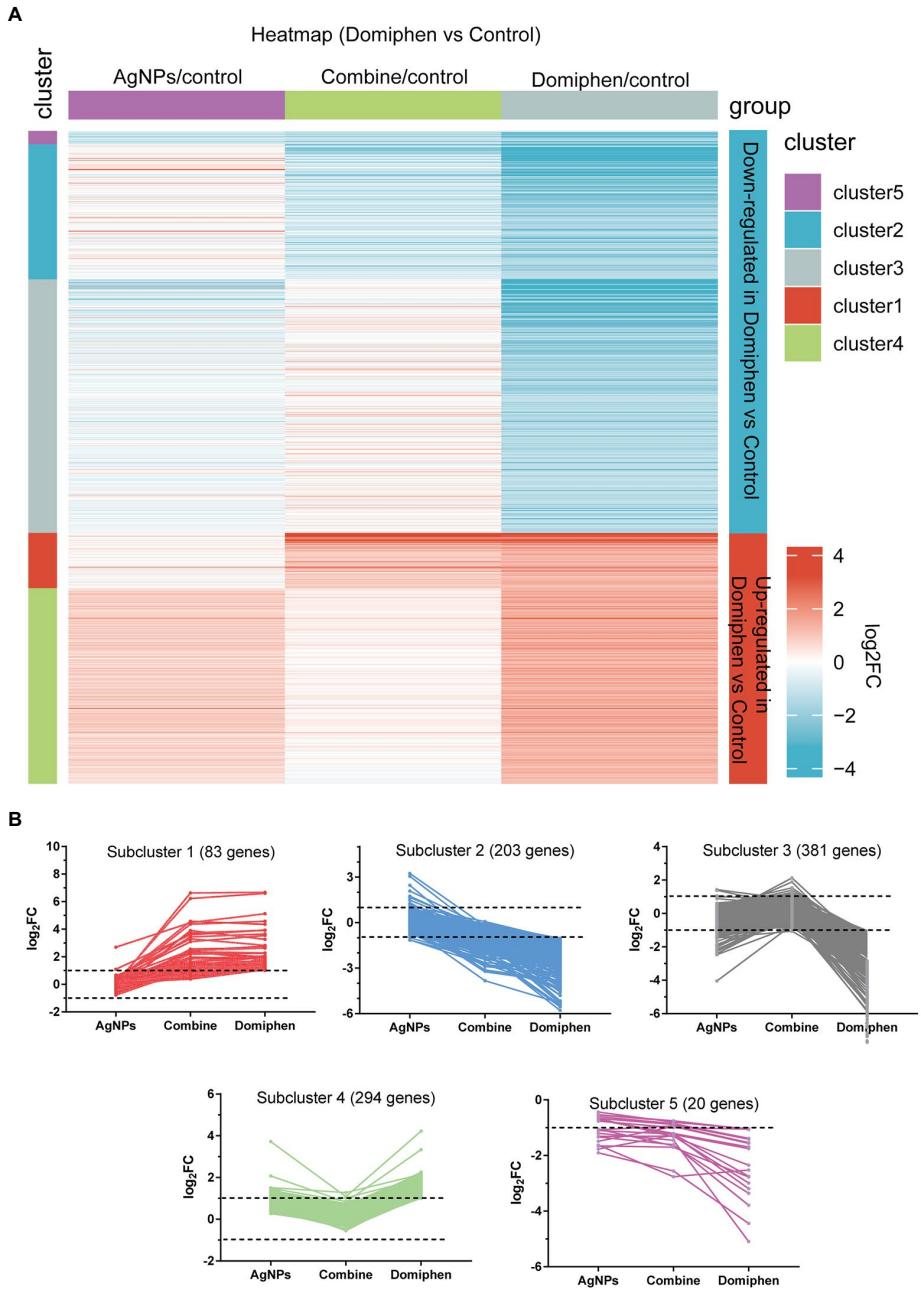
Transcriptional response of *A. fumigatus* to domiphen. **(A)** Heatmap showing the changes in expression of the genes significantly downregulated (blue; the upper) and upregulated (red; the bottom) after 1 h treatment of domiphen. Each column represents a group, and each row represents a DEG. The color represents the expression value (log_2_FC, the average from three parallel experiments) of the gene in each group. Red and blue represent log_2_FC > 0 and log_2_FC < 0, respectively. “1–5” indicate that multiple expression clusters could be differentiated. **(B)** The average expression (log_2_FC) of multiple genes.

**Figure 3:**
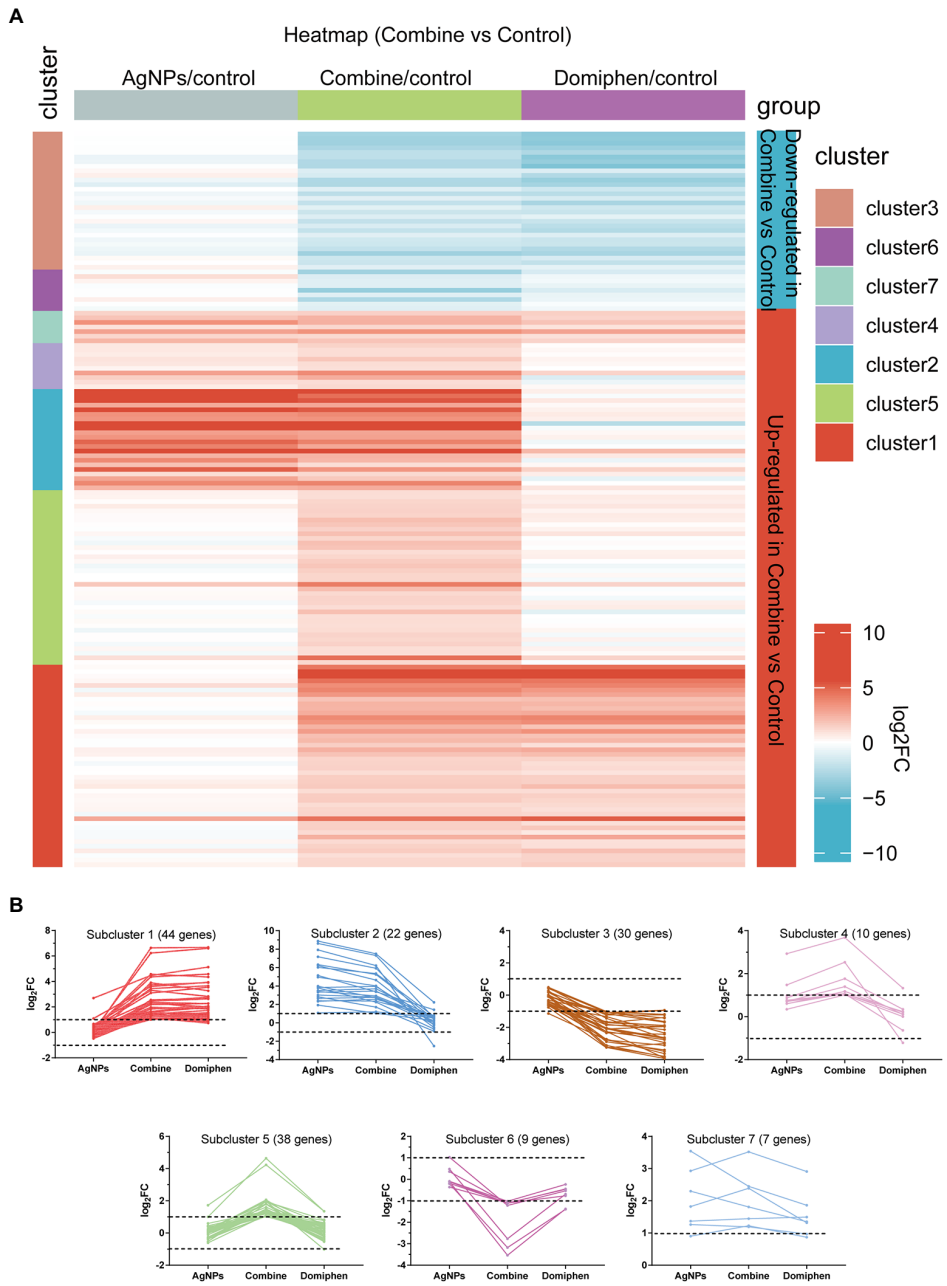
Transcriptional response of *A. fumigatus* to AgNPs combined with domiphen. **(A)** Heatmap showing the changes in expression of the genes significantly downregulated (blue; the upper) and upregulated (red; the bottom) after 1 h treatment of AgNPs combined with domiphen shown as “Combine.” Each column represents a group, and each row represents a DEG. The color represents the expression value (log_2_FC, the average from three parallel experiments) of the gene in each group. Red and blue represent log_2_FC > 0 and log_2_FC < 0, respectively. “1–7” indicate that multiple expression clusters could be differentiated. **(B)** The average expression (log_2_FC) of multiple genes.

To identify DEGs (differential expression genes) between two different groups, the expression level of each gene was calculated according to the transcripts per million reads (TPM) method. RSEM-v1.3.3^4^ was used to quantify gene abundances (Li and Dewey, 2011). Essentially, differential expression analysis was performed using the DESeq2/DEGseq/edgeR/Limma (Robinson et al., 2010; Wang et al., 2010; Love et al., 2014; Ritchie et al., 2015), DEGs with |log_2_ (fold change)| ≥ 1 and P-adjust ≤0.05 (DESeq2/edgeR/Limma) were considered to be significantly different expressed genes. In addition, we performed functional enrichment analysis, GO (Gene Ontology^5^) and KEGG (Kyoto Encyclopedia of Genes and Genomes^6^), to determine compared with the whole-transcriptome background, which DEGs were significantly enriched in top 20 GO terms at P-adjust ≤0.05 and KEGG pathways at *p*-value ≤0.05. GO functional enrichment and KEGG pathway analysis were performed by Goatools-v1.2.4^7^ and KOBAS-v2.0^8^ (Xie et al., 2011). Clustering heatmaps were created using the package “ComplexHeatmap-v2.13.1” in software R-v4.2.1 and Euclidean distance calculation (Gu et al., 2016). In the selected gene set (significantly changed genes under AgNPs, domiphen and combine conditions for Figures 1–3, respectively), clustering was performed with log_2_FC under all tested conditions. Through repeated iterations, we calculated the relative distance between genes using the Euclidean distance algorithm with average linkage and divided the selected gene set into different subclusters according to the relative distance of genes.

### 2.11. Constructions for PPI

We used STRING database^9^ for protein interaction network analysis of genes. The corresponding interaction relationship of genes was directly extracted from the database to construct the network. Then, networkX under Python was used for network visualization of genes.

### 2.12. Cytotoxicity test

HT22 cells were spread in 96-well plates at a density of 3–5 × 10^4^ cells/ml, 100 μl per well, and placed in a cell culture incubator (37°C, CO_2_ level of 5%). HT22 cells were incubated with 1 μg ml^−1^ of AgNPs, 2 μg ml^−1^ of domiphen or 0.25 μg ml^−1^ of AgNPs and 0.5 μg ml^−1^ of domiphen given after cell apposition. At the end of incubation, the medium was aspirated and washed 2–3 times with PBS. One hundred micro liter of incomplete medium containing 10 μl of CCK-8 assay solution (Cell Counting Kit-8; Abcam, England) was added and incubated for 1–2 h in an incubator protected from light. The OD value was measured at 450 nm. The absorbance of the experimental group was recorded as As using the following formula. The absorbance of the control group and blank group were recorded as Ac and Ab, respectively (Liu et al., 2017; Ou et al., 2021).


Survival rate of cells=(As−Ab)/(Ac−Ab)x100%

### 2.13. Statistical analysis

Data were expressed as Mean ± standard deviation (Mean ± SD), and Graphpad Prism 7 software (GraphPad Software Technology Co., Ltd., San Diego, CA) were used for statistical analysis. Two-tailed unpaired Student’s T test was used for comparison between the two groups. Comparisons between multiple groups were made using one-way ANOVA with a completely randomized design. The level of hypothesis test was determined by α = 0.05. *p* < 0.05 indicated statistically significant difference.

## 3. Results

### 3.1. Synergistic antimicrobial effects of AgNPs and domiphen on human fungal pathogen *Aspergillus fumigatus*

**Figure 4:**
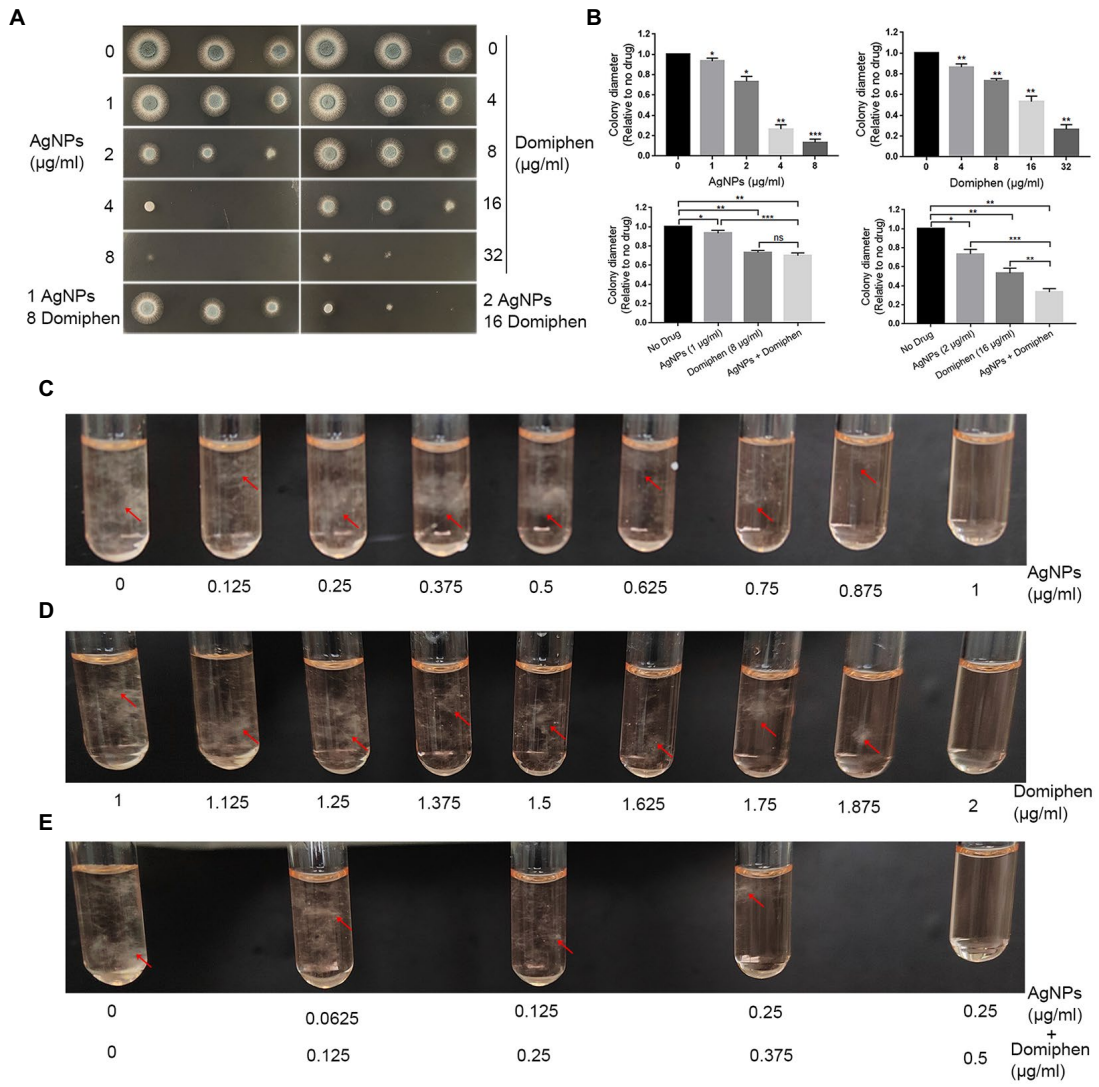
The single and combined susceptibility tests of AgNPs and domiphen against human fungal pathogen *A. fumigatus*. **(A)** Two microliters of double distill water containing 2 × 10^4^, 2 × 10^3^ or 2 × 10^2^ conidia of *A. fumigatus* wild-type strain (A1161) were used to inoculate onto YAG medium containing the indicated concentrations of AgNPs or domiphen. **(B)** Quantifications of colony diameter for the indicated strains under different treatment cultural conditions. *, *p* < 0.05; **, *p* < 0.01; ***, *p* < 0.001; ns, *P* > 0.05 compared to the no drug control or the single drug according to unpaired t test with or without Welch’s correction. **(C)** MIC (μg ml^−1^) test results of control group and indicated concentrations of AgNPs groups against *A. fumigatus*. **(D)** MIC (μg ml^−1^) test results of control group and indicated concentrations of domiphen groups against *A. fumigatus*. **(E)** MIC (μg ml^−1^) test results of control group and indicated concentrations of AgNPs combined with domiphen groups against *A. fumigatus*. For C to E, the mycelia were highlighted with red arrows.

In order to explore the antimicrobial effects of AgNPs and domiphen against *A. fumigatus*, we performed plate inoculation assays with different drug concentrations. As shown in Figure 4A, the growth of *A. fumigatus* was obviously inhibited in the presence of 2 μg ml^−1^ (0.0185 μmol ml^−1^) of AgNPs or 16 μg ml^−1^ (0.0386 μmol ml^−1^) of domiphen, suggesting that the antimicrobial effect of AgNPs against *A. fumigatus* is better than that of domiphen. To test the combined antimicrobial effects of AgNPs and domiphen against *A. fumigatus*, we also detected the growth of *A. fumigatus* under the two drugs. The results of combined drug susceptibility test showed that the colony diameter of *A. fumigatus* was much smaller under 2 μg ml^−1^ of AgNPs and 16 μg ml^−1^ of domiphen than that under corresponding concentrations of single drugs, indicating that the antimicrobial activity of combining AgNPs with domiphen was more active than that of the single drug (Figures 4A,B). The MICs of AgNPs and domiphen against *A. fumigatus* were determined by the microdilution method and the CLSI method (Espinel-Ingroff et al., 2011). As shown in Figure 4C, the solution was turbid when AgNPs was not added. When the concentration of AgNPs was 1 μg mL^−1^ or more, the growth of *A. fumigatus* was totally inhibited with no mycelia could be seen in the solution. Thus, the minimum inhibitory concentration (MIC) of AgNPs against *A. fumigatus* was 1 μg mL^−1^. Similarly, the MIC of domiphen against *A. fumigatus* was 2 μg mL^−1^ (Figure 4D and Table 1). To assess the joint effects of the two agents, combined drug susceptibility tests were performed. As shown in Figure 4E, the growth of *A. fumigatus* was totally inhibited under 0.25 μg ml^−1^ of AgNPs and 0.5 μg ml^−1^ of domiphen. The combined use of AgNPs and domiphen produced synergistic effects against *A. fumigatus* (FIC = 0.5, Table 1). Domiphen could increase the growth inhibitory activity of AgNPs against *A. fumigatus* in a planktonic and biofilm culture, resulting in a 4-fold decreased MIC of AgNPs (from 1 μg ml^−1^ toward 0.25 μg ml^−1^ in the presence of 0.5 μg ml^−1^ domiphen, Table 1). We also detected the antimiacrobial effects of AgNPs and domiphen in MM media (Supplementary Figure S1). The result showed that the MIC value in MM media is the same as that in RPMI-1640 media. Taken together, these results suggested that AgNPs, 2 μg ml^−1^ of domiphen and domiphen are synergistic antimicrobial against human fungal pathogen *A. fumigatus*. The two drugs combined could reduce the dose and toxicity of drugs compared to the drug alone.

**Table 1:** Drug susceptibility test for combination of AgNPs and domiphen with or without copper (1 mM CuCl_2_) or nitrogen source (1 mM ammonium tartrate).^**1**^

**Treatments**	**MIC (μg ml**^**−1**^**)**		**MIC’ (μg ml**^**−1**^**)**		**FIC**
	**AgNPs**	**Domiphen**	**AgNPs**	**Domiphen**	
**Control**	1	2	0.25	0.5	0.5
**Copper**	0.75	2	0.125	0.5	0.417
**Nitrogen source**	1.125	2	0.25	0.625	0.535

1 MIC, the MIC of single drug; MIC’, the MIC of combined drug.

### 3.2. AgNPs combined with domiphen have relatively low cytotoxicity

**Figure 5:**
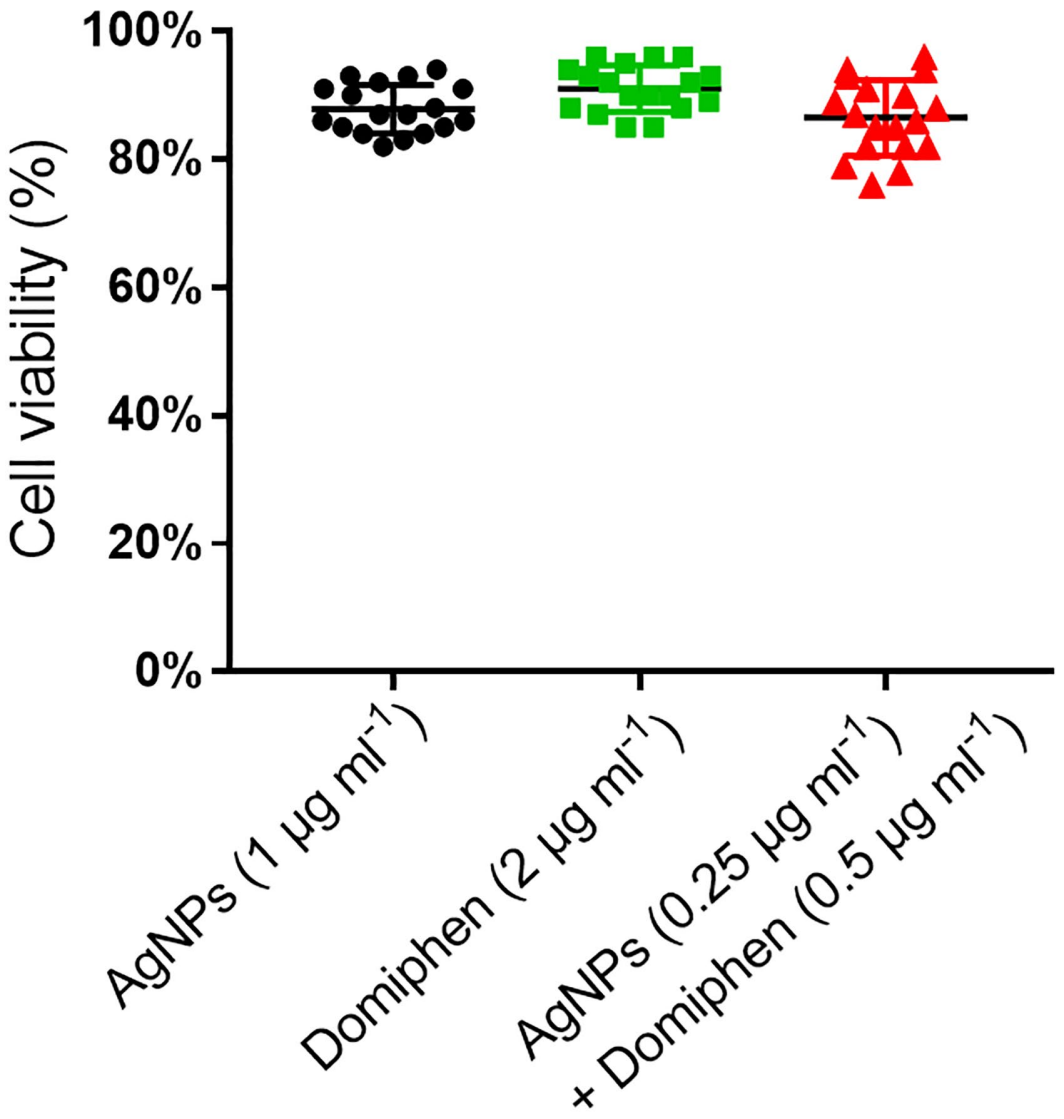
The HT22 cell viability of AgNPs (1 μg ml^−1^), domiphen (2 μg ml^−1^) and AgNPs (0.25 μg ml^−1^) combined with domiphen (0.5 μg ml^−1^), n = 18.

To monitor the cytotoxicity of AgNPs and domiphen, we performed cytotoxicity test. we chose nerve cells (HT22) instead of lung cells to perform cytotoxicity test for the following reasons: Firstly, considering that metal ions such as Ag + are more likely to cause neurotoxicity (Amadi et al., 2019), we are more concerned about the effect of AgNPs on the survival rate of nerve cells; Secondly, nerve cells are more fragile than lung cells, so a drug with low cytotoxicity for nerve cells is very likely to have low cytotoxicity for lung cells as well; Thirdly, although the primary sites of *A. fumigatus* infection are the lungs and paranasal sinuses, brain infection arises from direct invasion from the paranasal sinuses or by hematogenous spread (Kumar et al., 2021). There is also a case reporting invasive aspergillosis involving the lungs and brain in an immunocompetent person (Kumar et al., 2021). Due to the single and combined MICs of the two drugs were determined, we chose the corresponding MIC value of the two drugs as test condition. As shown in Figure 5, the cell viability under 1 μg ml^−1^ of AgNPs, 2 μg ml^−1^ of domiphen and 0.25 μg ml^−1^ of AgNPs combined with 0.5 μg ml^−1^ of domiphen conditions was about 87, 90 and 85%, respectively. This result suggested that AgNPs and domiphen have relatively low cytotoxicity.

### 3.3. Transcriptional profiles of *Aspergillus fumigatus* under AgNPs, domiphen, and AgNPs combined with domiphen conditions

**Figure 6:**
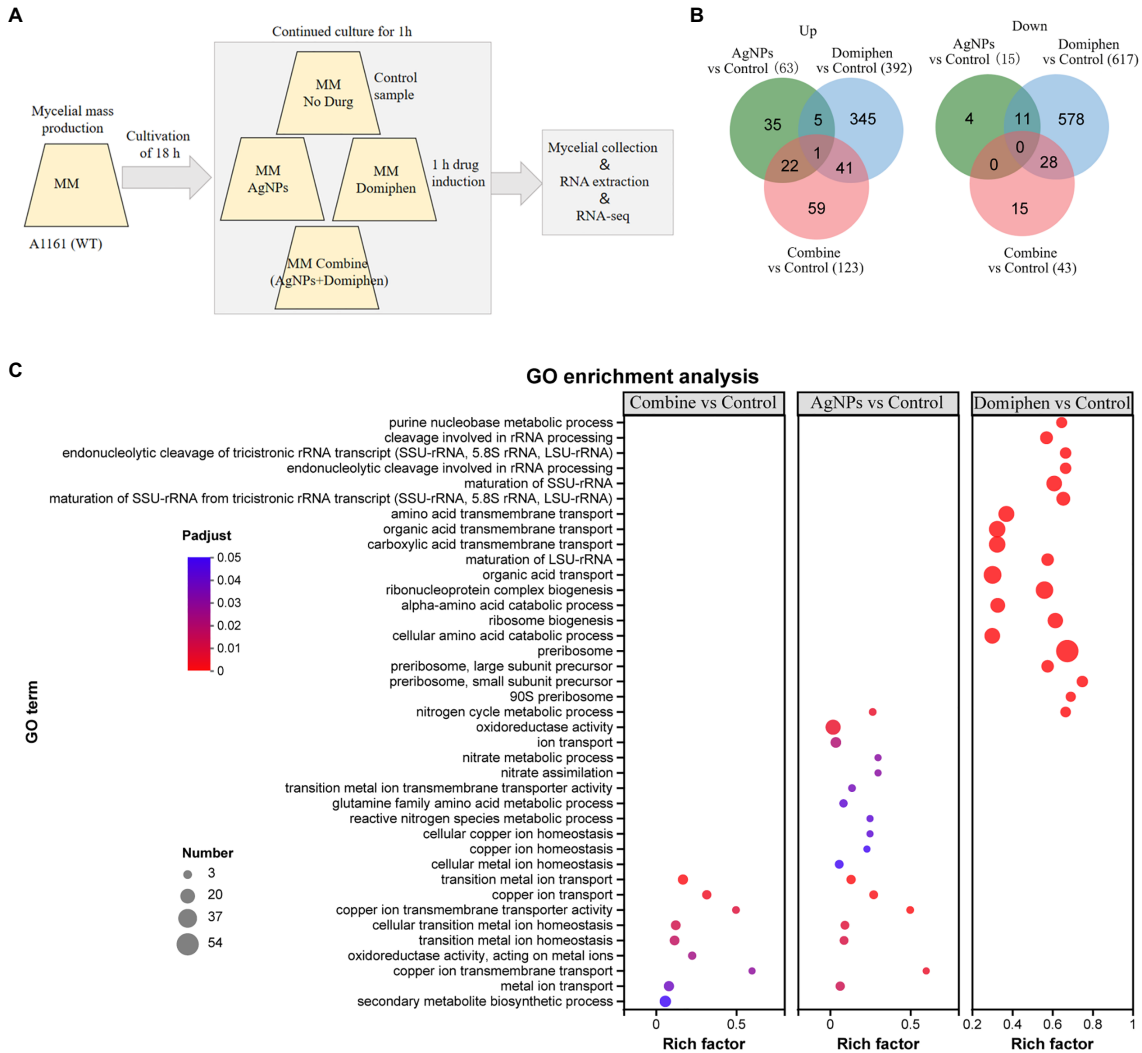
Overview of transcriptome of *A. fumigatus* under different drugs conditions. **(A)** Experimental design for the RNA-seq experiment carried out in this study. Conidia of wild-type strain were collected and incubated (3 × 10^5^ conidia ml^−1^) in MM for 18 h at 37°C. Then, AgNPs (1 μg ml^−1^), domiphen (2 μg ml^−1^), or AgNPs (1 μg ml^−1^) combined with domiphen (2 μg ml^−1^) indicated as “Combine,” were added, followed by an incubation of 1 h. Cultures grown in MM with no drug were used as controls. Samples (triplicates; see the Methods section) were then filtered and processed for RNA extraction and sequencing. **(B)** Venn diagrams showing the number of up-and down-regulated DEGs for different groups. **(C)** Bubble diagram of top 20 ranked GO terms of DEGs. The vertical axis indicates GO terms and the horizontal axis represents the Rich factor. The enrichment degree was stronger with a bigger Rich factor. The size of dots indicates the number of genes in the GO term.

To dissect the possible mechanism underlying synergistic antimicrobial effects of AgNPs and domiphen against *A. fumigatus*, we performed RNA-seq analyses comparing the *A. fumigatus* wild type with and without drug treatments. As shown in Figure 6A, conidia of wild-type strain were collected and incubated in MM for 18 h at 37°C. Then, AgNPs, domiphen, or AgNPs combined with domiphen were added, followed by an incubation of 1 h. Samples were then filtered and processed for RNA extraction and sequencing. As shown in Figure 6B, AgNPs was found to induce differential expression of 78 genes in *A. fumigatus*. Of these genes, 63 were up-regulated and 15 were down-regulated in AgNPs-treated *A. fumigatus* wild-type strain compared with untreated control (*p* < 0.05, |log_2_ (fold change)| ≥ 1, Supplementary Table 1). Domiphen was found to induce differential expression of 1,009 genes in *A. fumigatus*. Of these genes, 392 were up-regulated and 617 were down-regulated in domiphen-treated *A. fumigatus* wild-type strain compared with untreated control (*p* < 0.05, |log_2_ (fold change)| ≥ 1, Supplementary Table 2). The combination of AgNPs and domiphen was found to induce differential expression of 166 genes in *A. fumigatus*. Of these genes, 123 were up-regulated and 43 were down-regulated in combine-treated *A. fumigatus* wild-type strain compared with untreated control (*p* < 0.05, |log_2_ (fold change)| ≥ 1, Supplementary Table 3). These results suggested that domiphen has a greater impact on the transcription level of *A. fumigatus* compared with AgNPs.

To shed some light onto the functional roles of the DEGs, GO enrichment analysis was conducted. As shown in Figure 6C and Supplementary Table 4, “copper ion transmembrane transport” occupied the strongest enrichment degree as it had the highest Rich factor (0.6), followed by “copper ion transmembrane transporter activity” (Rich factor 0.5) in the AgNPs-treated *A. fumigatus* wild-type strain compared with untreated control. The over-expressed genes enriched for biological processes and pathways that have been implicated in copper ion transmembrane transport, copper ion transport, cellular copper ion homeostasis, metal ion transport and ion transport. The down-regulated genes enriched for biological processes that have been involved in nitrate assimilation (Supplementary Table 4). These results suggested that AgNPs inhibits the growth of *A. fumigatus* by enhancing metal ion transport, especially copper ion and suppressing nitrate assimilation. In the domiphen-treated group (Figure 6C; Supplementary Table 5), the top 5 ranked GO terms of DEGs are ‘preribosome, small subunit precursor’ (Rich factor 0.75), ‘90S preribosome’ (Rich factor 0.69), ‘preribosome’ (Rich factor 0.675), ‘endonucleolytic cleavage of tricistronic rRNA transcript’ (Rich factor 0.67) and ‘endonucleolytic cleavage involved in rRNA processing’ (Rich factor 0.67). As shown in Supplementary Table 5, the up-regulated genes enriched for biological processes and pathways that have been implicated in endonucleolytic cleavage of tricistronic rRNA transcript, maturation of SSU-rRNA from tricistronic rRNA transcript, ribosome biogenesis, maturation of rRNA and cleavage involved in rRNA processing. The down-regulated genes enriched for biological processes that have been involved in purine nucleobase metabolic process and amino acid transmembrane transport. These results suggested that domiphen could activate the transcript, processing and maturation of rRNA, and could inhibit purine nucleobase metabolic process and amino acid transmembrane transport to produce antimicrobial effects. Taken together, AgNPs and domiphen could inhibit the growth of *A. fumigatus* by suppressing nitrate assimilation, and purine nucleobase metabolic process and amino acid transmembrane transport, respectively.

Interestingly, the combine-treated *A. fumigatus* and the AgNPs-treated *A. fumigatus* had a similar GO enrichment pattern, which is different from that of the domiphen-treated *A. fumigatus* (Figure 6C; Supplementary Table 6), suggesting that when the two agents are combined, AgNPs has epistatic effects on domiphen. Specifically, the up-regulated genes enriched for biological processes and pathways that have been implicated in copper ion transmembrane transport, transition metal ion transport, iron ion transport and cellular transition metal ion homeostasis. The down-regulated genes enriched for biological processes that have been involved in secondary metabolite biosynthetic process. These results suggested that combination of AgNPs and domiphen could creat a low-copper condition with high expression of high affinity copper transporter and inhibit secondary metabolic process to combat *A. fumigatus*.

**Figure 7:**
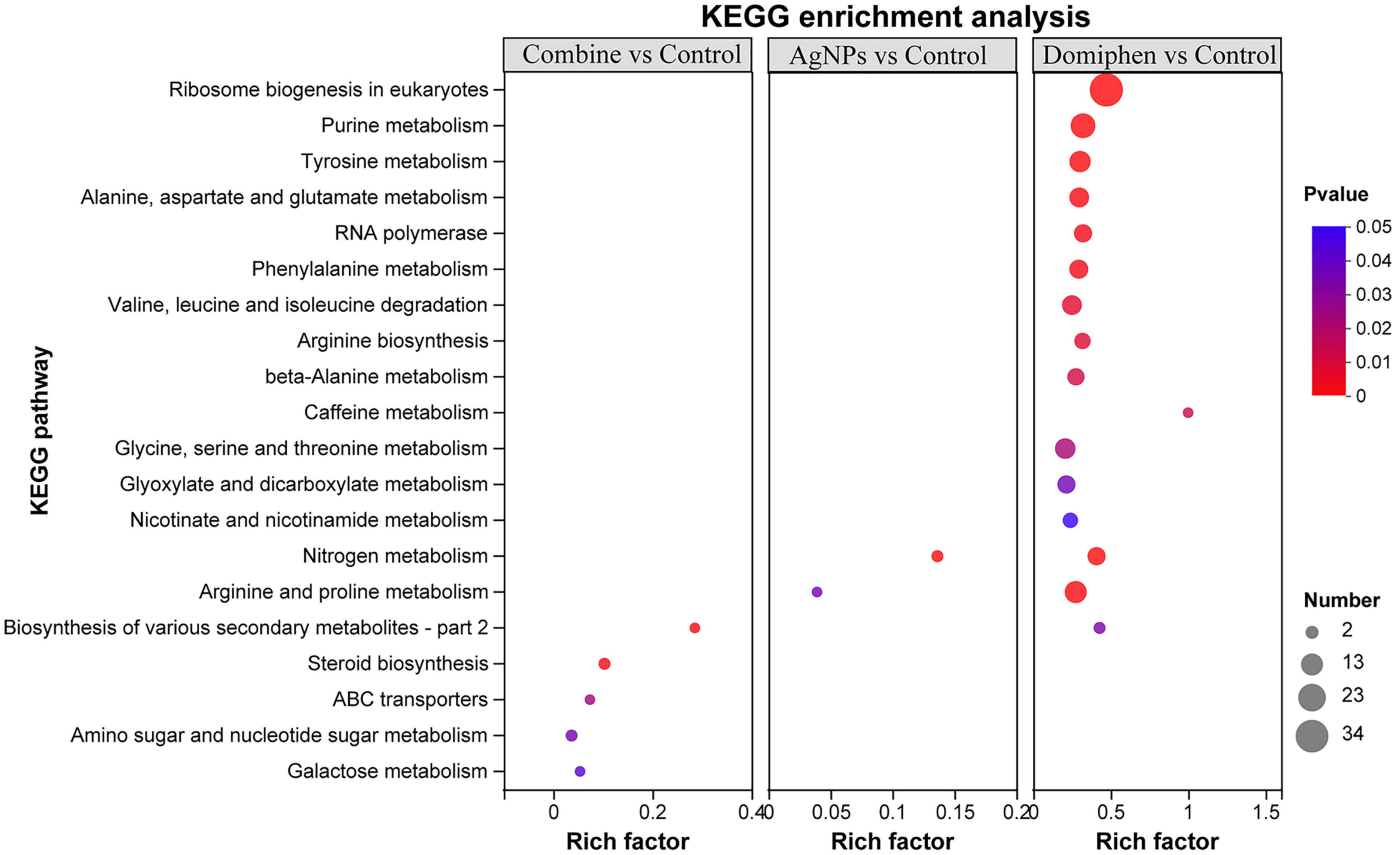
Bubble diagram of top 20 ranked KEGG pathways of DEGs among indicated groups. The vertical axis represents the pathway name, and the horizontal axis represents the ratio of the number of enriched gene/transcript samples in this pathway to the number of background annotated genes/transcripts. The larger the Rich factor, the stronger the enrichment degree. The size of the dots indicates the number of genes in the pathway, and the color of the dots corresponds to different Padjust ranges.

To further dissect the functional roles of the DEGs, KEGG enrichment analysis was performed. As shown in Figure 7 and Supplementary Table 7, ‘nitrogen metabolism’ possessed the highest rich factor (Rich factor 0.14) and had the most number of DEGs in the AgNPs-treated group, followed by ‘Arginine and proline metabolism’ (Rich factor 0.039), suggesting that AgNPs could mainly suppress nitrogen and amino acid metabolism to inhibit the growth of *A. fumigatus*. In the domiphen-treated group, ‘caffeine metabolism’ possessed the highest rich factor (Rich factor 1) and ‘ribosome biogenesis in eukaryotes’ had the most number of DEGs (Figure 7; Supplementary Table 8), indicating that domiphen could enhance ribosome biogenesis and suppress caffeine metabolism to inhibit the growth of *A. fumigatus*. For the combine-treated group, ‘biosynthesis of various secondary metabolites’ possessed the highest rich factor (Rich factor 0.29). ‘Steroid biosynthesis’ and ‘amino sugar and nucleotide sugar metabolism’ had the most number of DEGs (Figure 7; Supplementary Table 9), demonstrating that the combination of AgNPs and domiphen possibly influence secondary metabolites biosynthesis, steroid biosynthesis and nucleotide sugar metabolism to have a synergistic antimicrobial effect on *A. fumigatus*.

### 3.4. AgNPs-inducing transcriptomics changes in *Aspergillus fumigatus*

The heatmap in Figure 1A includes all the genes up-and down-regulated under AgNPs conditions in the wild-type strain, but was built considering their expression values for all conditions tested. The upper contains genes that are downregulated and the lower contains genes upregulated under AgNPs condition (blue and red, respectively). Multiple gene expression patterns could be differentiated (Figure 1B; Supplementary Table 10). Highlighted are five main expression clusters among upregulated genes and one among downregulated genes under AgNPs conditions. Subcluster 1 and 2 contain genes upregulated under AgNPs and “Combine” conditions but almost unchanged under domiphen conditions, suggesting that these genes are AgNPs-dependent responsive. For instance, the high affinity copper transporter (AFUB_020800) belongs to subcluster 1 (Supplementary Table 10), suggesting that the high affinity copper transporter (AFUB_020800) could be induced under AgNPs condition regardless of domiphen. Subcluster 3 and 6 contain genes upregulated exclusively under AgNPs conditions, indicating that these 6 genes upregulate in a AgNPs-dependent manner. Subcluster 4 contains 7 genes upregulated under all conditions, implying that these genes could respond under all tested conditions. Subcluster 5 contains 12 genes downregulated under AgNPs and domiphen conditions but almost unchanged under “Combine” condition, demonstrating that AgNPs and domiphen could repress these genes expression to inhibit the growth of *A. fumigatus*. As shown in Supplementary Table 10, subcluster 5 mainly takes part in nitrogen compound metabolic process and transmembrane transport. These results suggested that AgNPs and domiphen could inhibit the growth of *A. fumigatus via* blocking nitrogen compound metabolic process and transmembrane transport.

### 3.5. Domiphen-inducing transcriptomics changes in *Aspergillus fumigatus*

Five gene expression clusters are highlighted in the heatmap in Figure 2A, with three of them corresponding to the downregulated genes and the remaining two corresponding to the upregulated genes (Figure 2B; Supplementary Table 11). Among upregulated genes, subcluster 1 contains 83 genes, the majority of which upregulated under “Combine” and domiphen conditions but not changed under AgNPs conditions compared to untreated control (Figure 2B), suggesting that these genes are domiphen-dependent responsive; Subcluster 4 contains 294 genes, the majority of which upregulated under domiphen and AgNPs conditions but almost unchanged under “Combine” conditions, indicating that part of these genes could respond to domiphen or AgNPs induction. Among downregulated genes, subcluster 2 contains 203 genes, the majority of which downregulated under “Combine” and domiphen conditions but unchanged under AgNPs conditions. Subcluster 3 contains 381 genes, the majority of which downregulated exclusively under domiphen condition, demonstrating that domiphen could suppress the expression of these genes to inhibit the growth of *A. fumigatus*. For example, the nitrate related genes belong to subcluster 3 (Supplementary Table 11), suggesting that domiphen could repress nitrate metabolism. Subcluster 5 contains 20 genes, the majority of which downregulated under all drug conditions compared to untreated control, suggesting that these genes are affected under all tested conditions.

### 3.6. AgNPs and domiphen-inducing transcriptomics changes in *Aspergillus fumigatus*

The heatmap in Figure 3A includes all the genes up-and down-regulated under AgNPs combined with domiphen conditions in the wild-type strain, but was built considering their expression values for all conditions tested. The upper contains genes that are downregulated and the lower contains genes upregulated under AgNPs combined with domiphen conditions (blue and red, respectively). Multiple gene expression patterns could be differentiated (Figure 3B; Supplementary Table 12). Highlighted are five main expression clusters among upregulated genes and two among downregulated genes under “Combine” conditions. Among downregulated genes, subcluster 3 and 6 contain genes, some of which downregulated under “Combine” and domiphen conditions but not changed under AgNPs conditions, suggesting that domiphen could decrease the expression of these genes to inhibit the growth of *A. fumigatus*. Among upregulated genes, subcluster 1 contains 44 genes upregulated under “Combine” and domiphen conditions but not changed under AgNPs conditions, indicating that these genes are domiphen-dependent responsive; Subcluster 2 and 4 contain genes, the majority of which upregulated under “Combine” and AgNPs conditions but unchanged under domiphen conditions, suggesting that these genes are AgNPs-dependent responsive; A prominent example of the high affinity copper transporter (AFUB_020800) belongs to subcluster 2 (Supplementary Table 12), implying that AgNPs rather than domiphen could induce the expression of the high affinity copper transporter. Subcluster 5 contains 38 genes upregulated exclusively under “Combine” conditions, demonstrating that these genes could only respond to the combination of AgNPs and domiphen; Subcluster 7 contains 7 genes upregulated under all drug conditions compared to no drug control, suggesting that these genes could respond to all drugs tested.

### 3.7. The network of PPI of DEGs

**Figure 8:**
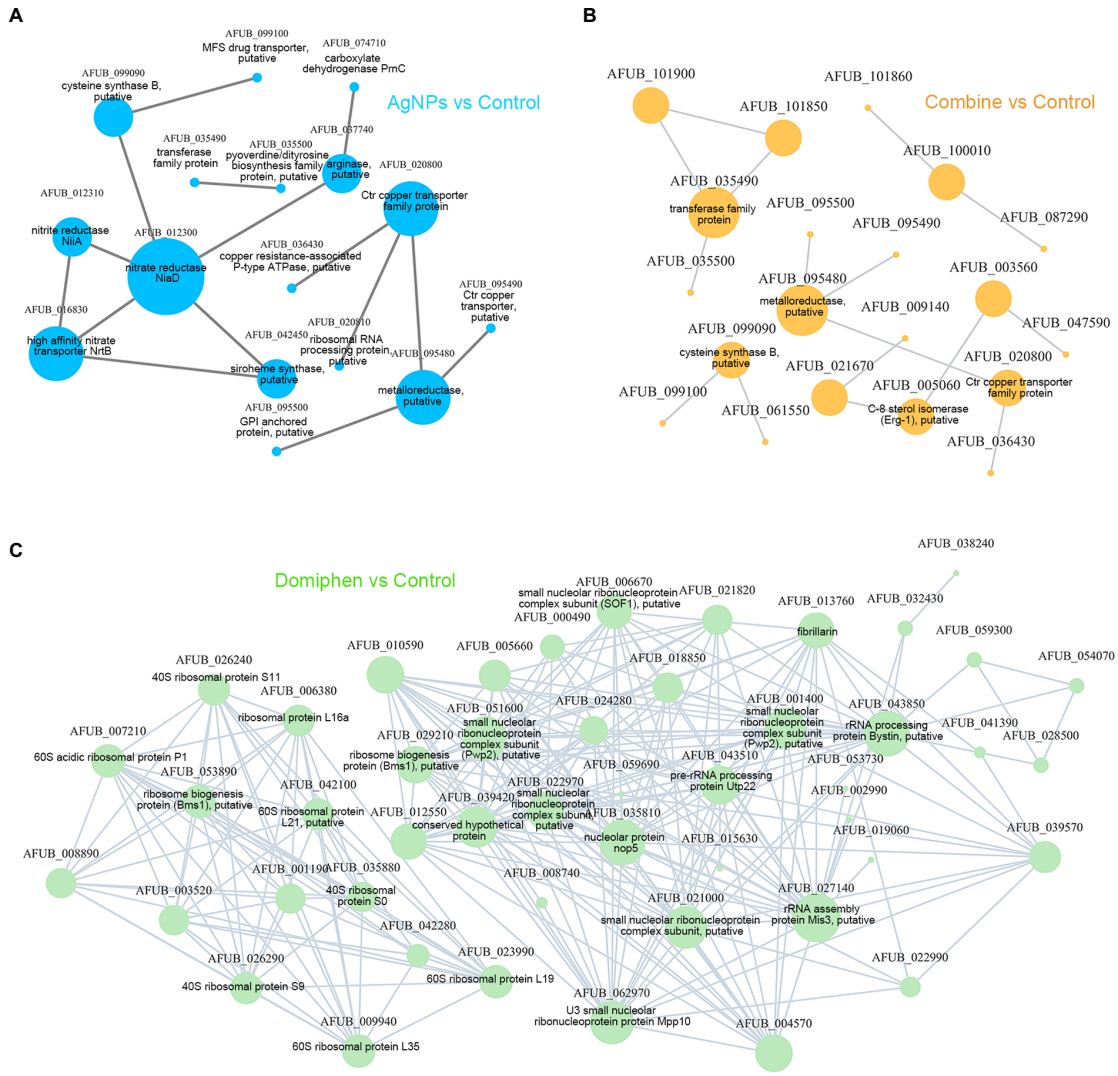
The network of PPI for DEGs. **(A)** The PPI analysis of DEGs in AgNPs vs. Control. **(B)** The PPI analysis of DEGs in Combine vs. Control. **(C)** The PPI analysis of DEGs in Domiphen vs. Control. The gene annotations come from the website EnsemblFungi: http://fungi.ensembl.org/index.html. Nodes represent genes/transcripts, and edges represent interactions between two genes/transcripts.

To further explore the possible mechanism in synergistic antimicrobial effects of AgNPs and domiphen against *A. fumigatus*, the putative 78 key DEGs belonging to the AgNPs-treated group, 1,009 DEGs belonging to the domiphen-treated group and 166 DEGs belonging to the combine-treated group were used to build PPI networks. As shown in Figure 8A and Supplementary Table 1, NiaD (nitrate reductase NiaD, AFUB_012300, fold change = 0.255), NrtB (high affinity nitrate transporter, AFUB_016830, fold change = 0.061) and CtrC (Ctr copper transporter family protein, AFUB_020800, fold change = 64.498), followed by AFUB_095480 (metalloreductase, putative, fold change = 6.319), had highest scores for betweenness centrality, indicating that they are most important for connections with other proteins and AgNPs could suppress nitrogen uptake together with the induction of copper transporters to inhibit the growth of *A. fumigatus*; Under domiphen conditions, the key proteins of *A. fumigatus* which were affected by domiphen are shown in Figure 8C and Supplementary Table 2, including small nucleolar ribonucleoprotein complex subunit (AFUB_022970, fold change = 3.219), rRNA processing protein Bystin (AFUB_043850, fold change = 2.15), 60S acidic ribosomal protein P1 (AFUB_007210, fold change = 2.521), etc. These results suggested that domiphen could enhance ribosomal protein biogenesis and rRNA processing to exert antimicrobial effects; Similarly, Figure 8B and Supplementary Table 3 showed that under the conditions of AgNPs combined with domiphen, the key proteins mainly affected are transferase family protein (AFUB_035490, fold change = 16.152), metalloreductase (AFUB_095480, fold change = 6.784) and CtrC (Ctr copper transporter family protein, AFUB_020800, fold change = 40.893), suggesting that the combination of AgNPs and domiphen could creat a low-copper condition with high expression of high affinity copper transporter and enhance transferase family protein synthesis to have synergistic antimicrobial effects.

### 3.8. Validation of the influence of nitrogen source and copper in the antimicrobial activity of AgNPs and domiphen

Considering that AgNPs and domiphen could inhibit nitrogen metabolism and AgNPs could enhance copper transporter expression, we want to investigate the role of nitrogen source and copper in the antimicrobial activity of AgNPs and domiphen. For this purpose, we performed drug susceptibility test under exogenous addition of nitrogen source or copper conditions. We first determined that the MIC of copper (CuCl_2_) was about 10 mM and nitrogen source (ammonium tartrate) had not antimicrobial effects. Thus, we chose 1 mM CuCl_2_ and ammonium tartrate which have no influence on the growth of *A. fumigatus* to conduct the subsequent experiment. As shown in Table 1, The MIC values of AgNPs were decreased under addition of copper condition, implying that copper could increase the antimicrobial activity of AgNPs and therefore improve the synergistic antimicrobial effects of AgNPs and domiphen (FIC = 0.417). On the contrary, nitrogen source could elevate the MIC values of AgNPs and domiphen (Table 1), implying that nitrogen source may abolish the synergistic antimicrobial effects of AgNPs and domiphen (FIC = 0.535). Taken together, these results suggested that copper and nitrogen source have opposite effects on the synergistic antimicrobial activity of AgNPs and domiphen.

## 4. Discussion

One of the most challenging global health threats is the development and spread of rapid microbial resistance to antibiotics that leaves infections untreatable, increasing the risk of surgery or immunosuppression for other diseases (Brogan and Mossialos, 2016). Many studies have shown that the potential of Ag as part of the solution to the antimicrobial resistance crisis (Abram and Fromm, 2020). Nano-antimicrobials are proving to be of particular value in preventing wound dressings or local infections. Therefore, this study provides insights into the mechanism of AgNPs combined with the potentiator domiphen as a potential strategy to combat the human pathogenic fungus *A. fumigatus*. Understanding the mode of action of antifungal compounds or combinations is important because it could lead to faster discovery of new antifungal therapies for the treatment of IA (Scorzoni et al., 2017). Currently, the commonly used antifungal drugs include caspofungin, voriconazole, amphotericin B, itraconazole, etc. (Ben-Ami and Kontoyiannis, 2021). We previously found that the combination of AgNPs and domiphen had synergistic antibacterial effects on *Acinetobacter baumannii* (FIC = 0.1875), *Staphylococcus aureus* (FIC = 0.5), *Escherichia coli* (FIC = 0.1875) and *Candida albicans* (FIC = 0.3125) (Hu et al., 2021). However, there is no relevant research on the synergistic antimicrobial mechanism of AgNPs combined with domiphen against the pathogenic fungus *A. fumigatus*. The research provides a theoretical basis and a new strategy for the treatment of IA.

In order to determine whether AgNPs and domiphen have antimicrobial effects on *A. fumigatus*, plate inoculation test was used to show that AgNPs and domiphen have good antimicrobial effects on *A. fumigatus*. To accurately determine the antimicrobial activity of AgNPs and domiphen alone on *A. fumigatus*, we also referred to CLSI standards and performed the single drug susceptibility test of AgNPs and domiphen against *A. fumigatus*. The results showed that the MICs of AgNPs and domiphen to *A. fumigatus* were 1 μg ml^−1^ and 2 μg ml^−1^, respectively. The above experimental results indicated that both AgNPs and domiphen could play good antimicrobial effects on *A. fumigatus*, but the antimicrobial effects of the combination of AgNPs and domiphen on common pathogenic fungus needs to be further verified. Therefore, in order to verify the combined antimicrobial effects of the two drugs, the checkerboard microdilution method was used to measure the combined drug susceptibility of AgNPs and domiphen at different concentrations. The results showed that the combination of AgNPs and domiphen had synergistic effects on *A. fumigatus* (FIC ≤ 0.5). Taken together, AgNPs and domiphen have obvious synergistic antimicrobial effects on opportunistic pathogen *A. fumigatus*, which could enhance the effectiveness of antimicrobial drugs with reduced dosage and toxic side effects of drugs.

In order to explore the combined antimicrobial mechanism of the two agents, RNA-seq was used in this study. Through GO enrichment analysis, KEGG enrichment analysis and cluster analysis, the gene sets that affects the growth of *A. fumigatus* could be accurately obtained when AgNPs, domiphen or combined agents are used. GO enrichment analysis together with the drug susceptibility test under addition of copper and nitrogen source conditions showed that AgNPs inhibits the growth of *A. fumigatus* by inducing high affinity copper transporter and suppressing nitrate assimilation; Domiphen could activate the transcript, processing and maturation of rRNA, and could inhibit purine nucleobase metabolic process and amino acid transmembrane transport to produce antimicrobial effects. When the two agents acted simultaneously, it was obvious that AgNPs had a greater effect on the growth process of *A. fumigatus* than domiphen. KEGG enrichment analysis showed that AgNPs could mainly suppress nitrogen metabolism to inhibit the growth of *A. fumigatus*; Domiphen could enhance ribosome biogenesis and suppress caffeine metabolism to inhibit the growth of *A. fumigatus*, while AgNPs combined with domiphen could affect the synthesis of secondary metabolites, steroid synthesis and material transport during the growth of *A. fumigatus*. It has been reported that Ag^+^ and AgNPs mainly interact with membrane proteins (Abram and Fromm, 2020), which may be the true reason why AgNPs could suppress nitrogen assimilation which relies on the membrane nitrate transporters. In line with our hypothesis, exogenous addition of nitrogen source could abolish the synergistic antimicrobial effect, suggesting that the synergistic antimicrobial effect partially relies on the nitrogen source deficiency. On the one hand, Ag^+^ interferes with electron transport and ATP production by substituting protons in the sulfhydryl group, blocking the bacterial respiratory chain (Abram and Fromm, 2020). As a result, cell metabolism is suppressed, which is consistent with our data that AgNPs combined with domiphen could inhibit the metabolic processes of *A. fumigatus* such as nitrogen metabolism. On the other hand, the bacterial ribosome is one of AgNPs’ main intracellular targets (Yamanaka et al., 2005). Denaturation of ribosome results in the inhibition of amino acid metabolism by AgNPs. Domiphen could increase the permeability of membrane due to its surfactant properties (Tits et al., 2020), thereby causing AgNPs easily to enter cells, which could be one of the reasons why domiphen could be a potentiator of AgNPs. In addition, Jana Tits, et al. found that domiphen increases intracellular miconazole availability by enhancing azole import and possibly by releasing sequestered miconazole (Tits et al., 2021). Similarly, domiphen could probably affect the intracellular AgNPs distribution to increase the antimicrobial activity of AgNPs, which remains to be determined. Moreover, in line with our data that AgNPs and domiphen have low cytotoxicity (Figure 5), the safety of AgNPs or domiphen at relatively low doses *in vivo* had been confirmed by various documents (Tits et al., 2020; Xu et al., 2020; Zielinska et al., 2020). Thus, the combination of AgNPs and domiphen has the potential to be developed into a next-generation fungicidal therapy for the treatment of patients with IA.

The key proteins of *A. fumigatus* analyzed by PPI corresponded to the pathways in enrichment analysis, and it was very important to note that the transmembrane transport process of copper ion was one of the most influential factor for the growth of *A. fumigatus* under the action of AgNPs combined with domiphen on the basis of high expression level (40-fold) of copper transporter. Copper ion (Cu), regarded as an important virulence factor in fungal pathogens, is one of the essential elements in organisms, which affects many biochemical reactions in organisms and participates in various metabolic pathways (Festa and Thiele, 2011; Cai et al., 2017). Intracellular copper balance is important for the survival of microorganisms (Cai et al., 2017; Li et al., 2019). In the process of copper ion uptake, the high affinity copper transporter family is responsible for the transport of copper ions across the membrane under low-copper condition. Ctr copper transporter family protein (AFUB_020800) is one of the key proteins affecting this pathway (Cai et al., 2017). The expression of Ctr copper transporter could increase about 40-fold under AgNPs combined with domiphen conditions (Supplementary Table 3), suggesting that *A. fumigatus* may encounter a low-copper situation with induction of high affinity copper transporter, which may be one of the reasons for the synergistic antimicrobial effects of AgNPs and domiphen. The induction of high affinity copper transporter is just a response mechanism of *A. fumigatus* to low-copper condition. Our validation experiment also confirmed that copper could increase the synergistic antimicrobial effect, suggested that more Cu^+^ in cells are attributed to the induction of copper transporters under this copper-treated condition, probably resulting in copper toxicity. In accordance with the present findings, previous studies have demonstrated that AgNPs could influence murine copper metabolism in a size-dependent manner (Skomorokhova et al., 2020). However, there are some limitations should be noted. The data were mainly analyzed by bioinformatics on the basis of RNA-seq, and the potential functional enrichment of other genes should be further investigated.

In conclusion, our study verified that AgNPs and domiphen had antimicrobial activity against *A. fumigatus* when they were used alone. On the basis of combined drug susceptibility test and cytotoxicity test, it was found that the combined use of AgNPs and domiphen could play synergistic antimicrobial effects with low cytotoxicity. RNA-seq was used to find out the related pathways affected by the two drugs, and the specific reasons for the influence of AgNPs and domiphen on the growth of *A. fumigatus*. It provides a theoretical basis and a new strategy for the treatment of IA.

## Data availability statement

The original RNA-seq data have been deposited in the NCBI Sequence Read Archive under BioProject ID PRJNA856787 (https://www.ncbi.nlm.nih.gov/bioproject/?term=PRJNA856787).

## Author contributions

WD and YL: conceptualization, resources, supervision, project administration, funding acquisition, writing—review and editing. WD, RX, and ZH; methodology. WD, HY, and YG: software. WD, RX, and YL: validation. RX: formal analysis. WD and RX: investigation. XX: data curation. WD and RX: writing—original draft preparation. RX, ZH, HY, and YG: visualization. All authors have read and agreed to the published version of the manuscript.

## Funding

This research was funded by Natural Science Foundation of Basic Research Program of Jiangsu Province-Project of Youth Foundation (BK20220658) and Excellent Talents Research Foundation of Xuzhou Medical University (D2021042) to WD. This study was also supported by 2020 Quality Cultivation Project of the School of Life Sciences, 2021 Higher Education Reform Research Project of School of Life Sciences (2021-1) and Key Projects of Jiangsu University Student Innovation and Entrepreneurship Training Program (202010313002Z).

## Conflict of interest

The authors declare that the research was conducted in the absence of any commercial or financial relationships that could be construed as a potential conflict of interest.

## Publisher’s note

All claims expressed in this article are solely those of the authors and do not necessarily represent those of their affiliated organizations, or those of the publisher, the editors and the reviewers. Any product that may be evaluated in this article, or claim that may be made by its manufacturer, is not guaranteed or endorsed by the publisher.

## Footnotes

^1^ https://github.com/OpenGene/fastp

^2^ http://ccb.jhu.edu/software/hisat2/index.shtml

^3^ https://ccb.jhu.edu/software/stringtie/

^4^ http://deweylab.biostat.wisc.edu/rsem/

^5^ http://www.geneontology.org

^6^ http://www.genome.jp/kegg/

^7^ https://github.com/tanghaibao/Goatools

^8^ http://kobas.cbi.pku.edu.cn/home.do

^9^ http://string-db.org/

## Abbreviations

RNA: seq
RNA: sequencing
AgNPs: silver nanoparticles
MIC: minimum inhibitory concentration
FIC: fractional inhibitory concentration
IA: invasive aspergillosis
MM: minimal medium
RIN: RNA integrity number
OD: optical density
DEGs: differential expression genes
TPM: transcripts per million
GO: Gene Ontology
KEGG: Kyoto Encyclopedia of Genes and Genomes
PPI: protein–protein interactions
