# Supplementary material for: Transcriptomics-based investigation of molecular mechanisms underlying synergistic antimicrobial effects of AgNPs and Domiphen on the human fungal pathogen Aspergillus fumigatus
Source: Front Microbiol. 2023 Feb 1;14:1089267. doi: 10.3389/fmicb.2023.1089267 (PMC9928863; doi:10.3389/fmicb.2023.1089267)
Supplement: Supplementary file 1 [file Data_Sheet_1.PDF]

Supplementary Material

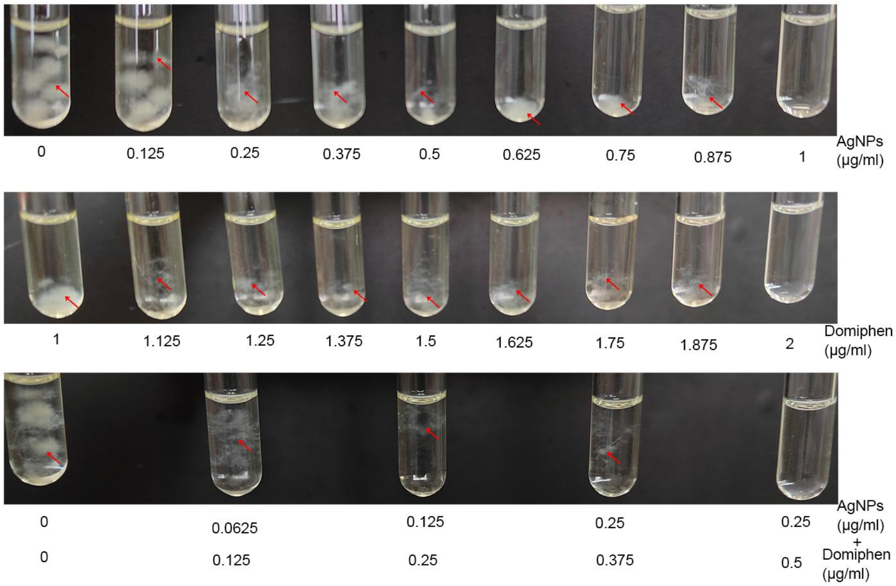

Supplementary Figure 1. MIC (μg ml^{-1}) test results of control group and indicated concentrations of agents against *A. fumigatus* in MM media. The mycelia were highlighted with red arrows.
